# Not Just Dyspnoea: Swallowing as a Concern for Adults with Laryngotracheal Stenosis Undergoing Airway Reconstruction

**DOI:** 10.1007/s00455-021-10287-3

**Published:** 2021-04-08

**Authors:** Gemma M. Clunie, Athina Belsi, Justin W. G. Roe, Caroline M. Alexander, Gurpreet Sandhu, Alison McGregor

**Affiliations:** 1 Imperial College London, London, UK; 2 Imperial College Healthcare NHS Trust, London, UK; 3 Sackler MSK Lab, Department of Surgery and Cancer, White City Campus, Imperial College London, 2nd Floor Michael Uren Hub, London, W12 0BZ UK

**Keywords:** Patients perspectives, Deglutition and deglutition disorders, Laryngotracheal stenosis, Dysphagia, Qualitative research

## Abstract

Acquired laryngotracheal stenosis (LTS) is a rare condition causing dyspnea and stridor. Patients often require multiple surgical procedures with no guarantee of a definitive outcome. Difficulty swallowing is a recognised problem associated with LTS and the reconstructive surgeries required to manage the condition. The breathlessness patient’s experience impacts on swallowing, and the vulnerable structures of the larynx are implicated during complex surgeries. This leads to dysphagia post-surgery, with some patients experiencing more chronic symptoms depending on the biomechanical impact of the surgery, or a pre-existing dysphagia. Despite this there is limited observational research about the dysphagia associated with LTS, with no exploration of the patient experience. Our aim was to investigate patient experience of living with LTS focussing on dysphagia in order to guide clinical practice. A qualitative study was completed using focus groups and semi-structured interviews with 24 patients who have had reconstructive surgery for LTS. Thematic analysis was used to identify three over-arching themes: The Physical Journey, The Emotional Journey and The Medical Journey. Key sub-themes included the importance of self-management and control, presence of symptoms, benefits of therapy, living with a life-long condition, fear and anxiety, autonomy, medicalisation of normal processes and the dichotomy between staff expertise and complacency. Swallowing was connected to all themes. The results are reviewed with consideration of the wider literature of lived experience particularly in relation to other chronic conditions and those that carry a high symptom burden such as head and neck cancer. Future clinical and research recommendations have been made. Akin to other clinical groups, adults with LTS are keen that management of their swallowing is person-centred and holistic.

**Supplementary Information:**

The online version contains supplementary material available at 10.1007/s00455-021-10287-3.

## Introduction

Acquired laryngotracheal stenosis (LTS) is a rare condition characterised by a narrowing of the airway from the supraglottic larynx to the carina [1]. This leads to dyspnoea, stridor, and in the most severe cases patients require a permanent tracheostomy. The causes of LTS are multifactorial and include intubation trauma, autoimmune disorders, idiopathic disease and radiotherapy [2]. Treatment for LTS includes repeated laser and dilatation procedures as well as more comprehensive reconstructive procedures such as laryngotracheal reconstruction (LTR) or tracheal and cricotracheal resections (CTR) [3].

Those patients who undergo reconstructive airway procedures have usually undergone multiple previous surgeries to manage their condition [4, 5] with repeated episodes of increased stridor and dyspnea often leading to attendance at an emergency department. Up to ten percent of patients are misdiagnosed with asthma and thus treated ineffectively with bronchodilators and steroids prior to their LTS diagnosis [6]. Definitive LTS treatment aims to improve symptoms of breathlessness and achieve decannulation, but this not always guaranteed [7, 8]. The uncertainty of diagnosis, treatment trajectory and outcome is extremely challenging for patients and, similarly to other chronic health conditions, has an impact on mental health as well as physical symptoms [9, 10].

In recent years, research has focussed on the functional impact of LTS and the complex reconstructive procedures necessary to manage the condition [11–13] alongside the primary outcome of improving the airway. There is increased acknowledgement that disruption of delicate upper airway structures and muscles can lead to dysphagia and dysphonia following airway surgery [12, 14]. During open reconstructive procedures a laryngofissure is required, the cartilaginous structures of the larynx are manipulated, and scar formation and contracture can occur to varying degrees. In some cases of airway resection the suprahyoid musculature is released [1]. This, along with the presence of the stent, can lead to swallowing problems post-surgery [3]. The interplay between airway, swallowing and voice is a key feature of LTS management, and the dyspnea caused by LTS can have a negative impact on swallowing, similarly to other respiratory conditions [15].

Existing studies on dysphagia as a primary outcome of LTS and airway reconstruction have explored data such as duration of tube feeding post-reconstruction [16] and a clinician-rated scale to assess changes in swallowing function before and after surgery [11]. The clinical presentation and best practice management of dysphagia in this population has been reported in both the adult and paediatric literature [17, 18]. There are no adult studies that have focussed on the biomechanical mechanism of dysphagia as a result of LTS or airway surgeries, however clinical experience shows that for the majority of patients significant dysphagia is short-term, and is related to a combination of reduced swallow efficiency and safety [17]. However, the anecdotal experience in our specialist Airways clinic is that many patients do report more prolonged mild dysphagia symptoms and some patients experience on-going swallowing difficulties due the dyspnea caused by LTS or repeated airway surgeries. This is particularly true for patients with supraglottic stenosis, where opening the airway above the larynx eliminates another level of airway protection for patients. Pre-existing dysphagia can also be a complicating factor, for example patients with co-occurring radiation-associated dysphagia alongside their LTS [3, 17].

The heterogeneity and rarity of LTS makes it challenging to determine healthcare impact and costs in the broad population. One study determined that the approximate added cost of tracheal injury, resulting from intubation damage, to a hospital stay was $1888 [19], without consideration of the multiple interventions subsequently required to manage the condition. Major reconstructive procedures show cost-effectiveness [20] but run the potential risk of other health-related complications, for example dysphagia, which carries its own cost implications [21]. This, combined with the acknowledged negative impact that both LTS and dysphagia can have on quality of life [10, 22], provides a clear rationale for better understanding the dysphagia experienced by LTS patients. Parental concerns relating to quality of life following paediatric airway reconstruction have been explored [23], however, to the best of our knowledge there are no studies in adults, within this population, looking at the patient experience of living with dysphagia or considering the overall impact of swallowing difficulties on patient’s lives.

Within the head and neck cancer literature there has been a focus on the lived experience of patients with dysphagia [24–26]. The relevance of this to the field of LTS research fits within the context of a long-term condition requiring complex treatment. These head and neck studies have shown that clinical practice can benefit from understanding patient perceptions and can lead to better quality care and service models [26]. Our aim in this study is to explore the patient experience of living with LTS with a focus on dysphagia. This understanding will help clinicians adjust their management and treatment of LTS patients in order to ensure their needs are being met as effectively as possible.

## Material and Methods

To investigate the lived experience of patients with LTS who undergo reconstructive surgery we conducted a qualitative study using a phenomenological methodology. This was chosen as it allows a systematic exploration of individual world views and how they have been impacted by their symptoms (for example dysphagia and dysphonia), illness and treatment [27] leading to a deeper understanding of the experiences and implications. A combination of focus groups and semi-structured Skype interviews were completed. Participants were recruited by directly from outpatient follow-up clinics at the only quaternary airway centre in the U.K. or indirectly by invitation letter if they had had an airway reconstruction at the centre. Participants were also recruited by patient partners via a social media group for patients with LTS. All patients approached had been treated under other laryngology services in the UK but the key inclusion criteria were that they had undergone an airway reconstruction procedure at our centre.

### Sampling

Purposive sampling was used to ensure that the full demographic range [28] of LTS patients was captured. This was necessary to comprehensively explore the heterogeneity of the condition and any co-existing dysphagia. Variation in the sample was achieved by identifying participants who met a pre-defined sampling criterion: gender (male/female), age, ethnicity, type of LTS, number of palliative surgeries for LTS, and definitive surgery for LTS. Dysphagia variables were not used in the sampling criteria as clinical details were not available for every participant approached. Due to the challenges of recruiting men into focus groups, the methodology was updated to include semi-structured interviews.

Recruitment started in December 2018 and continued until August 2019. Recruitment was stopped once inductive thematic saturation [29] was achieved, initially identified during the focus groups/interviews themselves, and confirmed with analysis of the data.

### Procedure

Ethical approvals were obtained, and all participants provided written informed consent. The principal investigator moderated each focus group and conducted the semi-structured interviews, with support from a second independent moderator for the first two groups. Both moderators had training in moderating and interviewing. The principal investigator was known to several of the participants as a clinician. Potential bias was mitigated by the use of reflexivity via a researcher diary. Each participant completed a demographic questionnaire that allowed them to self-describe their swallowing status including any current difficulties. A topic guide (see Online Appendix A) was used to pose questions to participants as well as focus discussions, but participants were encouraged to develop discussions and ensure that themes were fully elaborated within each group/interview. Use of the topic guide, and a structured moderation approach [30] ensured that group interaction was well controlled and remained purposeful. Focus groups lasted between 75 and 105 min and had between 3 and 5 participants. Semi-structured interviews were 40 min long.

### Data Analysis

The focus group and interview transcripts were analysed using thematic analysis according the process outlined by Braun and Clark [31]. Focus group transcripts were analysed at respondent level [32]. The principal researcher read transcripts several times to reach a basic understanding of participant’s experiences before beginning a process of inductive coding. This meant that themes and patterns were directly identified from raw data rather than prior assumptions [33]. Initial coding was completed using NVivo computer-assisted qualitative data assisted software (CAQDAS). Once transcripts had been coded and grouped into sub-sets and then themes by the principal researcher these were reviewed by a second investigator for consistency and agreement. The themes were then refined, and the transcripts reviewed and recoded using the final, highest level themes.

To ensure rigour the coding system and themes were reviewed by all authors, and by a patient advisory group who had helped design the research. They confirmed that the interpretation of the themes corresponded to their experience of LTS and reconstructive procedures. Themes were also reviewed by an independent researcher based in a separate institution to confirm reliability and minimise bias particularly in view of the dual role of the principal investigator as a researcher-clinician.

## Results

**Fig. 1:**
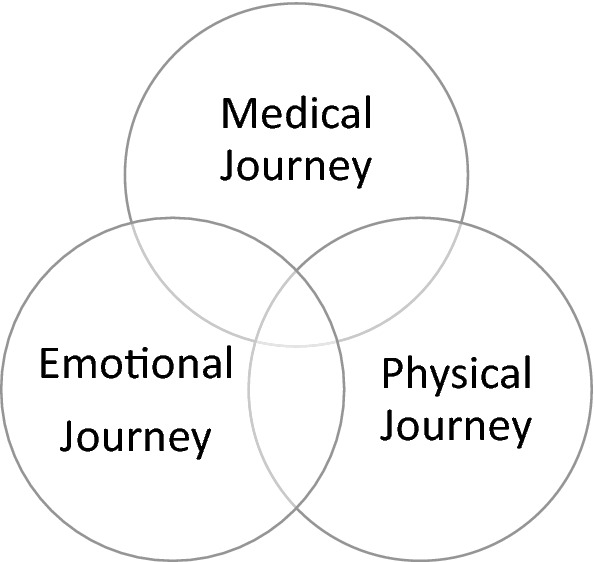
High level themes for the experience of Swallowing in LTS

**Fig. 2:**
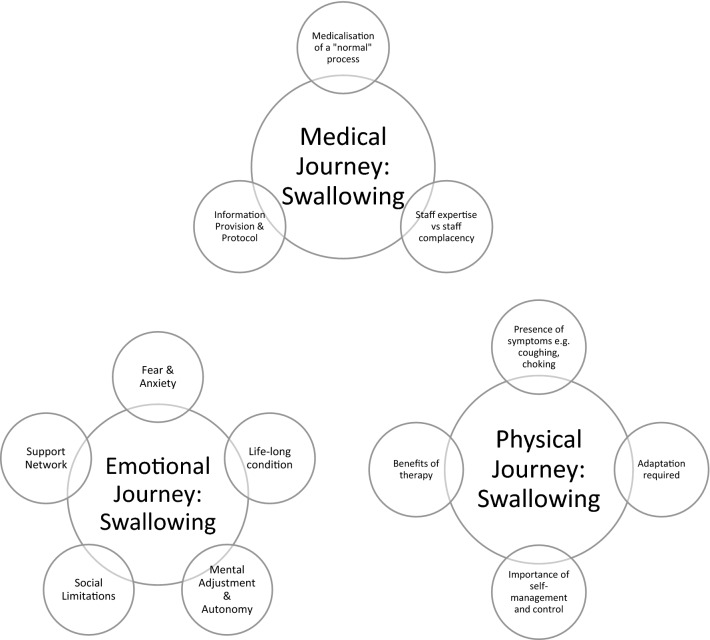
Sub-Themes of each high level theme

Of the 73 patients approached to participate in the study, twenty-four participants (33%) who had received reconstructive surgery for their LTS at the National Centre for Airway Reconstruction were recruited. Five focus groups with 22 participants and 2 semi-structured Skype interviews were completed between January 2019 and August 2019. Participant demographics are presented in Table 1. Analysis of responses from the focus groups and interviews revealed three overarching themes (see Fig. 1). The themes captured from the focus groups were consistent with those identified from the interviews. The first theme focussed on the physical experience of LTS and the surgery, symptoms and their management: *physical journey.* The second theme explored the impact of LTS on identity and its influence on social/personal and professional experiences: *emotional journey*. The last theme related to the experience of diagnosis, treatment and surgery as a patient: *medical journey*. Each of these themes encompassed multifactorial sub-themes (see Fig. 2).

**Table 1:** Demographics of participants (*n* = 24)

Demographic	Descriptor	No. of participants
Age	20–29	1
	30–39	5
	40–49	4
	50–59	6
	60–69	4
	> 70	4
Sex	Female	20
	Male	4
Ethnicity	White British	21
	White other	2
	Kenyan Indian	1
Cause of LTS	Intubation/tracheostomy	6
	Idiopathic	13
	Congenital	2
	Autoimmune	3
Number of surgeries for LTS	1–5	6
	6–10	5
	11–15	5
	16–20	5
	> 20	3
Definitive procedure for LTS	LTR	17
	Maddern	4
	CTR	2
	TR	1
Year of last reconstructive surgery	2002	1
	2012	1
	2013	2
	2014	2
	2015	2
	2016	4
	2017	6
	2018	5
Dietary level by time of group/interview (FOIS score)	7	17
	6	7

*LTS* laryngotracheal stenosis, *LTR* Laryngotracheal reconstruction, *CTR* cricotracheal resection, *TR* tracheal resection, *FOIS* functional oral intake scale

Whilst every participant acknowledged they would always “choose to breathe” and have reconstructive surgery, there was a unifying thread that other aspects of their lives were affected by that choice as well as the underlying condition. This paper focuses on swallowing and how this was experienced by patients with LTS and their management. Table 2 presents the self-reported symptoms of dysphagia from the study participants and Table 3 the available clinical details of dysphagia assessment for the cohort. Consistent with these findings and individual phenomenology, there was variability between participants in their experience of dysphagia. However, due to the use of a consistent topic guide all participants expressed opinions and attitudes that related to their personal experiences of swallowing. Each theme and sub-themes are supported by quotations from patients. These are presented with the code M/F to indicate gender (male/female), age and FG (Focus Group) or I (Interview) along with a number for each focus group (1–5), for example F, 45, FG3 or M, 27 I1.

**Table 2:** Patient reported dysphagia characteristics of cohort

Self-reported swallowing difficulties prior to reconstructive surgery	
Yes	6
No	18
Self-reported swallowing difficulties at time of focus group/interview	
Yes	14
No	10
Self-description of difficulties	
Chronic issue	12
Temporary during hospital admission	2
Choking	3
Dry, crumbly foods	2
Require extra chewing	1
Coughing	1
Need water when eating	2
Wary/anxious	1
Aspiration/residue	2

**Table 3:** Available details of dysphagia assessment for cohort

Dysphagia assessment	
None recorded	2
FEES	12
VFSS	9
Clinical evaluation of swallowing	1
Timing of intervention	
Inpatient	7
Outpatient	7
Both	8
PAS scores	
Pre-surgery	
8	2
3	1
1–2	3
Post-surgery	
8	3
5	2
4	1
3	3
1–2	1
Outpatient follow-up	
8	3
1–2	4

*PAS* Penetration-aspiration scale

### Theme 1: The Physical Journey

#### Presence of Symptoms and Adaptation

The physical experience of dysphagia was an acknowledged aspect of both LTS, and the reconstructive surgery required to manage the condition. Participants reported symptoms of choking, coughing and food sticking throughout the trajectory of their illness and treatment. The need to adapt how, when and what you eat in order to manage these symptoms was repeatedly referred to.

Depending on how many furball moments I have had in a day, is how I then have to think about what I am eating, and how I am eating. So, if I had had three or four already in the day I’ll think, ‘Actually, I’ll probably be alright if I eat my dinner now because I have shifted it,’ but if I haven’t had many furball moments then I know it is going to be a little bit more difficult.2 (F, 78, FG5)

#### Benefits of Therapy

For some participants these difficulties resulted in instrumentally identified dysphagia, particularly following surgical intervention. This required therapeutic assessment and management by Speech-Language Pathologists and was acknowledged as a positive experience.

I was still on tube feed after the operation…I kept having swallowing tests and doing a hundred tongue exercises a day and they worked! They really did. (F, 58, FG1)

#### Importance of Self-management and Control

However, a strong sub-theme of coping with dysphagia as part of the physical journey of LTS was the ability to be able to self-manage difficulties, without professional intervention. Unlike other symptoms, for example dyspnea, dysphagia was frequently rationalised despite potentially meeting the criteria of a disordered swallow. Participants defined swallowing difficulties in a covert way. They reported their ability to hide them with simple actions or behaviours. For example, they described the need to have water with them all the time to alleviate the sensation of dryness they experienced and mitigate the symptoms and associated difficulties with certain food groups.

It’s not a straightforward swallow. There’s definitely something which causes it to lodge. And then I have a drink of water and it does go. (M, 80, I2)

This sense of being able to manage the physical symptoms of dysphagia seemed important for participants as it was an aspect of their condition that they were able to exert a degree of control over. As one participant (F, 75, FG5), who had dysphagia therapy in the past but did not wish to continue with it, described “It’s making something [swallowing] that we mostly do naturally, to make it quite deliberate, isn’t it? It’s learning how to make things deliberate, that we already in the past have learnt, you know quite intuitively”.

### Theme 2: The Emotional Journey

#### Fear and Anxiety/Lifelong Condition

The emotional aspects of dysphagia were embedded within the psychosocial impact of living with LTS. Patients reported fear and anxiety that related to living with a rare, lifelong condition. They described the way their lives were negatively impacted. This included significant worries related to swallowing. For some participants the dysphagia associated with their dyspnea was a major cause of their distress:

Before I had my first reconstruction I could hardly breathe whatsoever. I couldn’t eat, because I was choking on everything and I literally thought I was dying basically. (F, 57, FG5)

#### Mental Adjustment and Autonomy

Other participants acknowledged the existence of dysphagia but used practical language that reframed and normalised their difficulties. For the majority they could live with their dysphagia symptoms without needing to disclose them to others as part of their condition or receive extra formal treatment. This indicated mental adjustment to the impact that dysphagia had on their lives:

After the reconstruction I had swallowing tests and I mean I can swallow, its fine. I just find sometimes it makes me cough if I, probably if I rush or drink something that goes down the wrong way. (F, 53, FG1)

Participants’ autonomy in managing the condition was a key sub-theme within the emotional journey. It demonstrates the importance of robust internal coping mechanisms to be able to live with LTS successfully. This was a key aspect to the experience of dysphagia for most participants. While they acknowledged an alteration in their swallowing compared to “normal,” they were able to reframe the experience so that it did not cause them emotional distress. This was supportive of the sense of control necessary when considering the physical aspects of the dysphagia.

And I got so fed up [of the puree menu], I knew I’d be alright if I could just eat some soup… in the end I wrote to the dietitian ‘I’m not daft, I’m not going to push it. The puree diet is awful, can I just have some soup. I’ve weaned a baby, I know what I’m doing, I’ll be alright’…And once it was officially on the wall then they’d give me soup. And then I could eat soup, I could eat ice cream and I could eat the middle of a baked potato, mashed up with tuna or cheese. Not a lot of it, but I could at least eat a little bit…so that was what I lived on for two weeks. But I didn’t have to have the feeding tube put back in. (F, 43, FG1)

#### Support Network

For other participants, their external support network was key to helping them cope with both their dysphagia and their LTS. They reported the importance of family and friends who understood their condition and had been part of their journey, as well as the safety net they presented.

I worry now that I might choke one day, that I can't get up what’s there, it’s a big lump and I can't get it out. And, I always say to my husband, ‘If I’m choking and it’s really not shifting, you are going to have to ring 999 very quickly, because there is no way I’m going to get this out (F, 60, FG5)

#### Social Limitations

There was also an acknowledgement of the limitations dysphagia and LTS placed on social activities and the altered routines that became necessary as a result. This included the choice to avoid eating out, or eating with strangers, because of worries associated with the stigma of dysphagia symptoms. This presented a limitation to life that was shared by both participants and their families.

Yeah, I mean, well, I have trouble with eating I mean I haven’t been into a restaurant …with the wife for many, many, many years because I daren’t go into a restaurant because if I start choking that’s it (M, 68, FG3)

### Theme 3: The Medical Journey

Most participants referred to the medical aspects of their LTS diagnosis and treatment, and the way their swallowing was managed as a result of this. A key context to this theme is the need for patients to have a nasogastric tube (NGT) inserted during surgery due to the impact on the swallowing mechanism. Formal swallowing assessment is completed in the days following surgery to allow patients to recommence oral intake as quickly, and safely as possible. However, for some patients a period of nil-by-mouth (NBM) is required. Sub-themes included the benefits and pitfalls of staff expertise, medicalisation of swallowing and the importance of information provision and explanations, for example in relation to surgical protocols and usual care.

#### Expertise Versus Complacency

Participants were relieved to be treated at a centre of excellence, by staff who had experience of LTS, but expertise also led to complacency and lack of information and compassion about the surgical protocol. This included key aspects of their care that related to management of their swallowing:

I didn’t know I was going to have a feeding tube. That was the first thing I remember, the only thing I remember waking up was being pushed forward on the ward so they could X-ray me to check that the feeding tube was down the right hole…I had a bit of a shock (F, 46, FG4)

#### Medicalisation

Participants discussed the medicalisation of eating and drinking, the need for graded return to oral intake following surgery, and the cautious approach of their clinicians. This was often felt to be unnecessary by participants who had no concerns relating to their ability to swallow:

The only thing I found absolutely awful was the food, well, I never had a problem with the swallowing and as soon as I could I got off that bloody liquid diet stuff…and then…after two days of soft food… I then went on to the harder stuff because I thought I can’t eat this [the modified diet] (F, 69, FG3)

Participants were frustrated by the lack of trust placed in them by healthcare professionals to make sensible choices in relation to dietary modification.

#### Information Provision and Protocol

A key sub-theme was that they were unaware or confused about the process of swallowing assessment and the clinical rationale for recommendations. Participants could recall being kept nil by mouth (NBM) following their surgery and waiting for swallowing tests but were often unsure about why; or which healthcare professionals were responsible.

I do remember a few people stood around trying to assess whether I could eat or not, but I can’t remember if they were doctors or speech therapists, and I think they gave me a yoghurt, or something, and just stood and watched me eat it. (F, 31, FG2)

Participants who had been provided with clearer information relating to surgical protocols and explanations of swallowing assessments and management expressed more satisfaction about their hospital experience, even if they had experienced more significant dysphagia symptoms.

## Discussion

The aim of this paper was to explore the lived experience of dysphagia for people with LTS, in order to guide clinical practice and care. The 24 participants all acknowledged that their experience of eating and drinking had been affected by their LTS and reconstructive surgery to varying degrees ranging from mildly altered function due to surgical intervention, through to dysphagia requiring a prolonged period of rehabilitation. These experiences were described within three broad themes encompassing the physical, emotional and medical journey of living with LTS. Participants described the physical symptoms of dysphagia, but also the more subtle changes to swallowing even once they had recovered. Alongside any diagnosed dysphagia, these issues led to emotional challenges and impacted on social lives. The importance of self-management, whether through internal mechanisms or external support structures was also reported. Participants also reported the varying benefits and limitations of information provision, medical protocols and processes of managing swallowing in relation to LTS and the reconstructive surgery.

The multifaceted nature of how patients with LTS reported their experience of dysphagia and managing swallowing correlates with previous research on the lived experience of dysphagia in other conditions, for example head and neck cancer [25, 34]. Swallowing is not experienced solely as a physical process and the emotional challenges of managing difficulties are often felt more profoundly than the symptoms themselves [35]. This has implications for treating clinicians, who need to provide holistic support and guidance, even if only as a safety net [36], to LTS patients whose swallow function is clinically safe and efficient.

The continuum of swallowing difficulties has also been investigated within paediatric LTS research. A parental questionnaire of feeding status following reconstructive surgery reported that 31% of children experienced feeding difficulties following their surgery [23]—this included difficulties such as taking time with meals and coughing intermittently as well as tube feeding. These parental experiences are consistent with changes described by participants in our study. Whilst many participants did not have a formal diagnosis of dysphagia, there were changes to the psychosocial aspects of their eating and drinking, with negative psychological consequences over time. This difference between clinical profile and personal experience needs to be mitigated by careful questioning from clinicians [35] and use of a person-centred approach to care [37]. This need to offer swallowing management programs that go beyond the physical symptoms is consistent with studies exploring the lived experience of dysphagia in a head and neck cancer population [24–26].

The importance of accurate, detailed and patient-centred information provision in relation to swallowing has been explored in healthcare literature [26, 38, 39]. These findings mirror the experiences of the participants with LTS. For many, the lack of preparation or explanation of standard protocols and procedures relating to the management of swallowing following airway reconstruction led to a negative, confusing or frustrating experience. Healthcare professionals need to be mindful of the importance of preparing LTS patients for treatment in a personalised and holistic way and avoid the risk of clinician expertise leading to complacency [40]. A potential way to achieve this would be look to models of care that use patients to co-design protocols and pathways, thereby avoiding the disparity between patient and clinician expectations [41].

Previous research has shown that for patients living with a chronic condition, self-management strategies are key to empower them to care for themselves autonomously [42]. Seeking normalcy as part of this strategy is a vital part of successful self-management [43]. This potentially explains why for participants it was important to be able to manage the physical and emotional manifestations of swallowing difficulties with normalising language and behaviours. Self-efficacy and control are crucial aspects to living with a long-term condition such as LTS and dysphagia. These co-exist alongside the need for external support, for example from partners or family members [43].

This study is the first qualitative investigation of the experience of dysphagia for patients with LTS and the findings are a starting point to review clinical pathways and care. However, all participants were recruited from the same service, and although purposive sampling was used to ensure demographic range, their treatment and experiences were only representative of that service. In the UK there are no other centres that offer complex reconstructive procedures, so UK-based LTS patients cared for elsewhere would not have had similar experiences, making experiential comparison difficult. However, it is to be expected that a cohort of LTS patients who had undergone more minor, endoscopic surgical procedures to manage their condition would have milder underlying disease [44], and be less likely to experience dysphagia as a result of this, or their surgeries.

The methodological alteration to complete semi-structured interviews as well as focus groups did lead to a slight increase in male participants, however the majority of participants were women. This has been shown to be a challenge when recruiting to qualitative studies in other literature [45], and needs to be acknowledged as a limitation of the study. Despite this the analysis of themes identified by men and women relating to dysphagia in our research did not demonstrate any significant differences.

Another limitation is that no attempt was made to classify the different clinical profiles of swallowing of each participant or stratify mild/moderate/severe dysphagia as a demographic feature which may have provided alternative findings. Due to the absence of other qualitative studies exploring the lived experience and dysphagia of LTS patients, generalisability of the findings has had to take place in the context of research from other clinical areas, for example, head and neck cancer. However, the strong thematic parallels noted between this study and other qualitative datasets demonstrates similarity between the experiences of LTS patients and other groups. LTS is a rare, chronic condition often requiring repeated surgeries and associated with a symptom burden similar to that reported in head and neck cancer [46]. Further research into the impact for LTS patients will hopefully demonstrate the need for long-term support in this population as well.

## Conclusion

This study has shown that the patient experience of swallowing in LTS is complex and multi-faceted despite being a lesser known aspect of the disease. Patients require supportive, person-centred care when managing their condition, irrespective of the severity of symptoms and benefit from the consideration of the psychosocial as well as physical aspects of dysphagia. The study demonstrates the need for further research focussing on dysphagia in LTS, both from the patient perspective, for example to explore swallowing difficulties over time and impact of multiple surgeries but also to understand the physiology and underlying causes of the swallowing difficulty.

## Supplementary Information

Below is the link to the electronic supplementary material.

Supplementary Information 1 (DOCX 32 kb)
