# Supplementary material for: Not Just Dyspnoea: Swallowing as a Concern for Adults with Laryngotracheal Stenosis Undergoing Airway Reconstruction
Source: Dysphagia. 2021 Apr 8;37(2):365–74. doi: 10.1007/s00455-021-10287-3 (PMC8948149; doi:10.1007/s00455-021-10287-3)
Supplement: Supplementary file 1 — Supplementary Information 1 (DOCX 32 kb) [file 455_2021_10287_MOESM1_ESM.docx]

**Appendix I: Topic schedule**

**Study title: What are the voice and swallowing concerns of adults with airway stenosis who have had reconstructive airway surgery?**

**Introduce the study and researchers, confidentiality, timing, aims of the focus group, and plans for future research –** for example *“I know there are lots of things you would like to talk about, but I will be bringing you back to focus on swallowing and voice. However, I promise that if there are burning issues/repeated topics that emerge from discussion that we cannot explore in detail now we will use these themes for further projects that you will be able to participate in if you are interested.”*

|  | Topic Areas |
| --- | --- |
| **a.** | **Experience of surgery**   1. What was your experience before the reconstruction?   *Examples to explore*  Discussion of outcomes: Airway/Breathing/Other  Understanding of complications: Swallowing/Voice/Other  Were risks discussed: Airway/Swallowing/Voice  Team involved prior to surgery: Doctor/CNS/ SLT/ Other   1. What was your surgical experience? |
| **b.** | **Swallowing**   1. How was your swallowing?   *Explore*  Chronology: Before surgery/ after surgery  Changes to diet: Consistencies/ tube feeding/ NBM  SLT Input: Assessments/Therapy  Contexts: Hospital/Home/Out and about  Support: Who/What  Was it helpful? Was it needed? |
| **c.** | **Voice**   1. How was your voice?   *Explore:*  Chronology: Before surgery/ after surgery  SLT Input: Assessments/Therapy  Contexts: Hospital/Home/Work/Telephone  Support: Who/What  Was it helpful? Was it needed? |
| **d.** | **Life now**   1. Any on-going problems with voice or swallowing? 2. Do you continue to receive SLT input? 3. Do you continue to rely on support from family and friends? 4. What impact have your swallowing/voice problems had on your family/work life? |
| **e.** | **The ideal world**   1. In hindsight, what would you have liked to know before surgery? 2. Thinking about yourself before surgery and your future self (i.e. now) what tips would you give to someone if they were coming for surgery next week? How should they prepare? 3. What would be the features of the ideal care pathway (for swallowing and voice?) |
| **f.** | **Clarification and Conclusion**   1. Is there anything that you haven’t mentioned that you think is important to bring up before we finish? 2. Is there anything else that you think I should know to help me understand your situation? 3. Is there anything you would like to ask me? |
|  | Thank you for your participation |
